# Effect of Muscle Cell Preservation on Viability and Differentiation of Hamstring Tendon Graft In Vitro

**DOI:** 10.3390/cells10040740

**Published:** 2021-03-27

**Authors:** Jin Kyu Lee, Sungsin Jo, Young Lim Lee, Subin Weon, Jun-Seob Song, Il-Hoon Sung, Tae-Hwan Kim

**Affiliations:** 1 Department of Orthopaedic Surgery, Hanyang University Hospital, Seoul 04763, Korea; jklee77@hanyang.ac.kr (J.K.L.); sungih@hanyang.ac.kr (I.-H.S.); 2 Institute for Rheumatology Research, Hanyang University, Seoul 04763, Korea; joejo0517@gmail.com (S.J.); mylime20@gmail.com (Y.L.L.); tnqls2808@gmail.com (S.W.); 3 Department of Translational Medicine, Graduate School of Biomedical Science and Engineering, Hanyang University, Seoul 04763, Korea; 4 Department of Orthopaedic Surgery, Gangnam JS Hospital, Seoul 06259, Korea; sjsos1999@hanmail.net; 5 Department of Rheumatology, Hanyang University Hospital for Rheumatic Diseases, Seoul 04763, Korea

**Keywords:** anterior cruciate ligament reconstruction, tendon-derived cells, muscle-derived cells, multiple differentiation

## Abstract

Muscle tissue is often removed during hamstring tendon graft preparation for anterior cruciate ligament (ACL) reconstruction. The purpose of the study was to test whether preservation of muscle remnants on a tendon graft is beneficial to the graft healing process following ACL reconstruction. Co-culturing of tendon-derived cells (TDCs) and muscle-derived cells (MDCs) was performed at various ratios, and their potential for cell viability and multilineage differentiation was compared to a single TDC cell group. Ligamentous and chondrogenic differentiation was most enhanced when a small population of MDCs was co-cultured with TDCs (6:2 co-culture group). Cell viability and osteogenic differentiation were proportionally enhanced with increasing MDC population size. MDCs co-cultured with TDCs possess both the ability to enhance cell viability and differentiate into other cell lineages.

## 1. Introduction

Anterior cruciate ligament reconstruction (ACLR) is one of the most commonly performed surgical procedures in the field of orthopedic sports medicine [1,2]. Ruptured ACLs do not heal spontaneously and, given their important biomechanical function, ligament reconstruction is often required to treat an unstable ACL-deficient knee [3]. Surgical techniques for ACL reconstruction have evolved in recent years; however, re-rupture, which occurs in 6–25% of young active patients, remains a concern [4,5]. Recent studies have promoted the use of autografts for ACL reconstruction for young active patients because of their superior healing capacity compared with allografts [6]. Among available autograft options, the hamstring tendon (semitendinosus tendon and gracilis tendon) is one of the most commonly used graft types for ACL reconstruction because of low harvest-site morbidity compared with the patellar tendon [7,8,9]. A tendon graft requires two critical biological healing processes for successful ACL reconstruction: (1) tendon-to-bone integration, and (2) intra-articular graft ligamentization [10,11,12,13,14,15,16,17,18,19,20,21]. Sufficient tendon-to-bone integration in the bone tunnel is essential for early rehabilitation and return to sports activities [15]. However, current evidence describes fromation of indirect healing, which is defined as Sharpey-like fibers connecting the tendon-to-bone interface [10,11,12,13]. The intra-articular portion of tendon grafts must mature and acquire ACL-like biomechanical properties [14,15]. Multiple studies have shown that the intra-articular portion of reconstructed tendon grafts need to remodel into a ligamentous ACL-like structure, which is often referred to as the “ligamentization” process [14,15,16,17,18,19]. Falconiero et al. [14] reported that ligamentization occurs over a 12-month period with peak maturation evident after one year. Sanchez et al. [15] reported that the grafts undergo a ligamentization process beyond one year and do not reach maturity until approximately two years after surgery.

There is no universal protocol on how to surgically prepare a hamstring tendon graft. However, most surgeons tend to use a tendon graft obtained surgically after separating the tendons from the remaining muscle tissue, but this surgical procedure is not based on any biological background in the literature [22]. Muscle has been identified as a valuable source of postnatal stem cells and it possesses the ability to differentiate into other cell lineages, thus, preservation of muscle tissue may be beneficial to promote certain biological healing processes required for tendon healing [22,23,24]. Accordingly, we performed experiments to test the hypothesis that muscle possesses a comparable potential for multilineage differentiation as tendon tissue, and, therefore, preservation of muscle remnants on a tendon graft is beneficial to the graft healing process following ACL reconstruction. To test the effect of muscle preservation on the tendon, co-culturing of tendon-derived cells (TDCs) and muscle-derived cells (MDCs) was performed at various ratios, and their potential for cell viability and multilineage differentiation was compared to a single TDC cell group.

## 2. Materials and Methods

### 2.1. Patients

This study was carried out in accordance with institutional guidelines and approval from the Ethics Committee of Hanyang University Hospital, with written informed consent from all subjects (IRB-2018-05-001). Tendon and muscle tissues were harvested from 13 patients (10 males, 3 females) with a mean age of 22.3 ± 1.93 years (range: 19–33 years) who underwent primary ACL reconstruction using autogenous hamstring (gracilis and semitendinosus) tendon grafts.

### 2.2. Isolation and Culture of Human TDCs and MDCs

The harvested muscle and tendon tissues were dissected in 3–5-mm pieces using scissors and were digested in Dulbecco’s modified Eagle medium-high glucose (DMEM-HG; SH30243.01; Hyclone, Logan, UT, USA) containing 1% antibiotic (penicillin/streptomycin; 1540–122; Gibco, Waltham, MA, USA); and 1 mg/mL type-1 collagenase (17100–017; Gibco) in a water bath at 37 °C for 6–8 h. The digested tissues were filtered with a 70-µm cell strainer (93070; SPL) and then isolated primary cells were grown with DMEM containing 10% FBS and 1% antibiotics. The first 1–2 cell passages were stored in liquid nitrogen for future experiments. For experiments in this study, passages 3–6 were generally used.

### 2.3. Co-Culture Assay

A direct contact co-culture of TDCs and MDCs was performed. Two individual groups and three co-culture groups were tested. The single-cell groups were a TDC group and an MDC group. TDCs and MDCs were co-cultured at different ratios to form three mixed-cell groups—7:1 co-culture group, 6:2 co-culture group, and 4:4 co-culture group—where tendon tissue always comprised the larger proportion of the graft. For each experiment, the total number and ratio of TDCs and MDCs in the each group were described in detail.

### 2.4. Flow Cytometry Analysis (FACS)

The tendon and muscle cells were washed with Perm/Wash buffer (554715; BD Biosciences, San Jose, CA, USA) and stained with Alkaline phosphatase (ALP; 327308; BioLegend, San Diego, CA, USA), CD11b (550019; BD Pharmingen, San Diego, CA, USA), CD34 (343607; BioLegend), CD45 (368511; BioLegend), CD59 (3044711; BioLegend), CD74 (326811; BioLegend), CD90 (328109; BioLegend), CD150 (323205; BioLegend), CD146 (361015; BioLegend), or CD164 (324805; BioLegend) for 30 min at 4 °C. Following staining, the cells were fixed using a fixation/permeabilization solution kit (554715; BD Biosciences) and washed with Perm/Wash buffer (554715; BD Biosciences). Stained cells were analyzed by flow cytometry (FACS Canto II; BD Biosciences) and data images were managed with Flowjo software (BD Biosciences; San Jose, CA, USA).

### 2.5. ALP Staining and Activity

ALP activity and staining methods and analyses have been previously reported [25,26]. All TDCs and/or MDCs were seeded in a 96-well plate and incubated for one day. The next day, the cells were stained with extracellular ALP (85L2, Sigma, St. Louis, MO, USA) and assessed using an intracellular ALP colorimetric assay kit (K412-500, BioVision, San Francisco, CA, USA) following the manufacturer’s protocol.

### 2.6. Cell Viability Using Water-Soluble Tetrazolium Salts (WST) Assay

TDCs, MDCs, and co-cultured cells were cultured at 4 × 10^3^ cells/well in a 96-well plate. The cell numbers in the groups were: TDC group, 4 × 10^3^ TDCs; 7:1 co-culture group, 3.5 × 10^3^ TDCs and 0.5 × 10^3^ MDCs; 6:2 co-culture group, 3 × 10^3^ TDCs and 1 × 10^3^ MDCs; 4:4 co-culture group, 2 × 10^3^ TDCs and 2 × 10^3^ MDCs; and MDC group, 4 × 10^3^ MDCs. On the indicated day, 10 μL of EZ-CYTOX (EZ-1000, DoGen) was added to each well. After incubation for one hour, absorbance at 450 nm was measured with a microplate reader (Thermo Fisher, Waltham, MA, USA).

### 2.7. Ligamentous Differentiation and Activities

For ligamentous differentiation, cells were stimulated with human platelet-derived growth factor-BB (PDGF-BB; 100-14B, PeproTech, Rocky Hill, CT, USA) according to the indicated duration and dose as described [27,28]. Briefly, stimulating DMEM containing 10% FBS, 1% antibiotics, and 25 μg/mL PDGF-BB was changed every three days. The ligamentous status of stimulated cells was assessed via picrosirius red staining (ab150681, Abcam, Cambridge, UK) and total collagen assay (K218, BioVision), according to the manufacturer’s instructions. RT-qPCR was performed to quantify the transcription level of the tendon/ligament-related extracellular matrix (ECM) genes including tenascin-C (TNC) and collagen type I and III [29,30].

### 2.8. Total Collagen Assay

A total of 8 × 10^4^ cells/well (tendon and/or muscle cells, as indicated) were seeded in six-well plates and then the cells were incubated in the presence of vehicle (distilled water) or PDGF-BB for 14 days before total collagen assay (ab222942, Abcam) was performed. After 14 days of incubation, the cells were washed with 1X PBS and harvested with a scraper. NaOH at 10 N was added and the cells were placed on a heat block at 120 °C for an hour after the tube was sealed with paraffin film. All processes followed manufacturing protocols.

The cells were treated as described above. On the indicated day, the cells were stained with Sirius red (ab150684, Abcam) and incubated at room temperature for one hour. The plates were washed with tap water and air-dried overnight. Stained wells were imaged with a Nikon eclipse Ti-U microscope (Nikon; Minato).

### 2.9. Osteogenic Differentiation and Activities

The indicated tendon and muscle cell numbers (total 8 × 10^3^ cells/well) were seeded in a 96-well plate and replaced by osteogenic differentiation medium. Osteogenic differentiation methods and analyses have been previously reported [31,32]. The differentiated cells were washed with 1X PBS and fixed with 10% formalin (HT501640, Sigma) for Alizarin Red S (ARS; A5533, Sigma), absolute ethanol (1.00983.1011, Merck, Kenilworth, NJ, USA) for hydroxyapatite (HA; PA-1503, Lonza, Basel, Switzerland), and absolute methanol (1.06009.1011, Merck) for Von Kossa (VON). Early osteogenic differentiation status was assessed by ALP staining. Late osteogenic differentiation status was assessed by ARS staining solution to detect calcification and hydroxyapatite (HA) and staining with 1% silver solution to detect mineralization. Afterward, stained wells were imaged with a Nikon eclipse Ti-U microscope (Nikon; Minato).

### 2.10. Chondrogenic Differentiation and Activities

As previously reported [33], cell numbers were adjusted for chondrogenic differentiation because at least 2 × 10^5^ cells were required for sufficient growth. The cell numbers in the groups were: TDC group, 2 × 10^5^ TDCs; 7:1 co-culture group, 1.75 × 10^5^ TDCs and 0.25 × 10^5^ MDCs; 6:2 co-culture group, 1.5 × 10^5^ TDCs and 0.5 × 10^5^ MDCs; 4:4 co-culture group, 1 × 10^5^ TDCs and 1 × 10^5^ MDCs; MDC group, 2 × 10^5^ MDCs. A total of 2 × 10^5^ cells (TDCs and/or MDCs, as indicated) in DMEM growth medium were centrifuged for three minutes at 1500 rpm in 15-mL conical tubes. The cells were resuspended in 1 mL of chondrogenic differentiation medium, centrifuged for 3 min at 1500 rpm, and placed in a CO_2_-enriched incubator. The cells containing chondrogenic medium were incubated for seven days. After seven days, chondrogenic pellets were transferred from the 15-mL conical tubes to round-bottomed 96-well plates and incubated for seven days. The chondrogenic medium was composed of serum-free Dulbecco’s modified Eagle medium-nutrient mixture F-12 (DMEM/F12; 11330-032, Gibco), 10% insulin-transferrin-selenium (ITS)+ premix tissue culture supplement (I3146, Sigma), 10 μM dexamethasone (D-2915, Sigma), 1 µM ascorbate-2-phosphate (49752, Sigma), 1% sodium pyruvate (11360-070, Gibco), and 10 ng/mL of transforming growth factor-beta 1 (TGF-β1; 100-21, Peprotech). Chondrogenic differentiation medium was changed every three days. After two weeks of incubation, pellets were fixed with 10% formalin (HT501640, Sigma), washed with 1X PBS (L0615-500, Biowest, Nuaillé, France), and transferred to 30% sucrose solution overnight. Pellets were drowned in cryocompound (4583, SAKURA, Tokyo, Japan) and cut into 14–20-µm sections with a cryostat (CM1850, Leica, Wetzlar, Germany). The pellet slices were placed on gelatin-coated slides and stained with safranin O (1446640250, Sigma) and toluidine blue (89640-25G, Thermo Fisher). Stained slides were imaged using a Nikon eclipse Ti-U microscope (Nikon; Minato).

### 2.11. RT-qPCR

Total RNA extraction and RT-qPCR were performed as previously described [25]. Each target gene’s expression was compared with GAPDH expression. Summarized expression values were averaged and changes in mean multiples were calculated. RT-qPCR was performed to quantify the transcription level of cartilage specific genes including SOX9, collagen type II (COL2), and aggrecan (ACAN). The primers used for PCR are described in the Table 1.

### 2.12. Statistical Analyses

Graph-Pad Prism 6.0 was used for data management and statistical analyses. Statistical significance was assessed using Wilcoxon matched-paired tests and the Mann–Whitney test. Values are presented as mean ± standard error of the mean (SEM) from at least three independent experiments. The level of statistical significance was indicated by asterisks (* *p* < 0.05, ** *p* < 0.01, *** *p* < 0.001).

## 3. Results

### 3.1. Basal Characteristics of TDCs, MDCs, and Co-Cultured Cells

**Figure 1:**
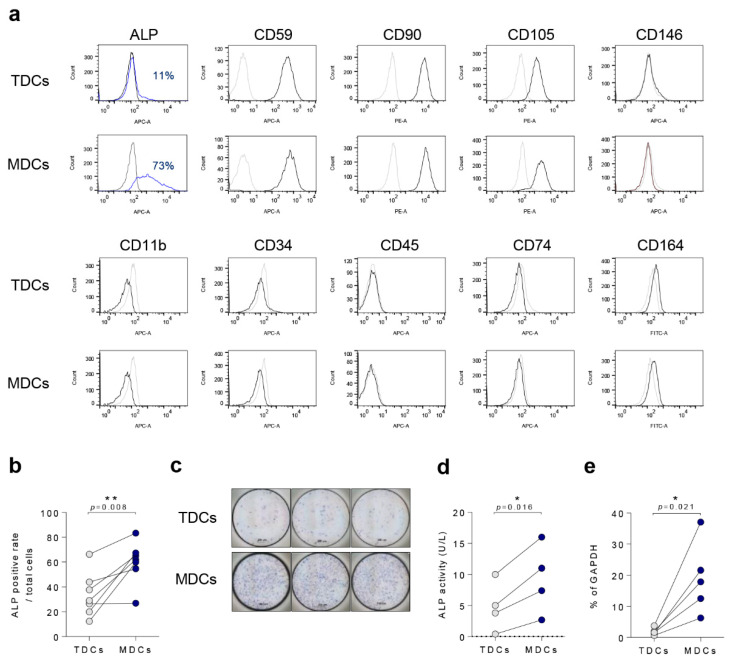
ALP was highly expressed in MDCs compared with TDCs. (**a**) Tendon and muscle surface markers such as ALP, CD59, CD90, CD105, CD146, CD11b, CD34, CD45, CD74, and CD164 were analyzed by FACS. (**b**) Quantification of surface ALP expression (n = 6). (**c**) All TDCs and MDCs were cultured in growth medium for a day and cells were assessed with ALP staining (scale bar = 200 µm, (**d**) ALP activity (n = 4), or (**e**) qPCR for ALP mRNA expression (n = 5). *, *p* < 0.05; **, *p* < 0.01. Data are presented as the mean ± SEM.

To distinguish specific surface markers between TDCs and MDCs, FACS analysis was performed. In both MDCs and TDCs, CD59, CD90, and CD105 were positively expressed, however CD11b, CD34, and CD45 markers were negative, indicating non-hematopoietic cell origins [34,35] (Figure 1a). Intriguingly, the ALP-positive cell population was larger (although not statistically significant) in the MDC group compared with the TDC group (Figure 1b,c), and ALP activity and relative mRNA expression levels were significantly higher in the MDC group compared with the TDC group (*p* = 0.016, and *p* = 0.021, respectively) (Figure 1d,e). In co-culture groups, ALP staining, activity, and relative mRNA expression were relatively higher than in the TDC group, but not significantly (Supplementary Figure S1a–c).

### 3.2. Cell Viability

**Figure 2:**
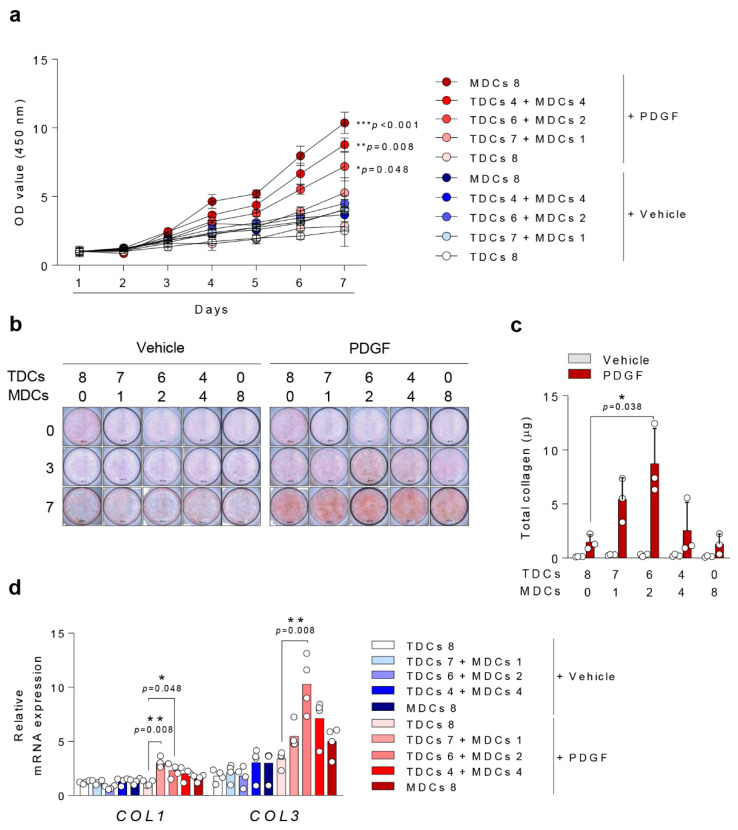
Platelet-Derived Growth Factors-BB (PDGF-BB/PDGF) potentiates ligamentous differentiation in TDCs with small population of MDCs. (**a**) A total of 4 × 10^3^ cells (TDCs and/or MDCs, as indicated) were cultured in growth medium for seven days and cell viability was measure using WST assay. A total of 8 × 10^4^ cells/well were seeded in six-well plates, stimulated with vehicle or PDGF-BB for 14 days, and analyzed by (**b**) Sirius red staining (scale bar = 200 µm) for collagen type 1 and 3, (**c**) total collagen assay, and (**d**) qPCR for collagen type 1 and 3 gene expressions. *, *p* < 0.05; **, *p* < 0.01; ***, *p* < 0.001. Data are presented as the mean ± SEM.

Cell viability was evaluated with WST assay. Across all evaluated days, the MDC group showed higher cell viability compared with the TDC group. On day seven, after PDGF-BB stimulation, the MDC group and co-culture groups (4:4, and 6:2 co-culture group) showed significantly higher cell viability than the TDC group, suggesting that MDCs possess a stronger capacity for cell viability (*p* = 0.008, *p* = 0.048) (Figure 2a).

### 3.3. Ligamentous Differentiation and Collagen Synthesis

All groups showed an increase in total collagen synthesis after PDGF-BB stimulation (Figure 2b). The 6:2 co-culture group showed significantly higher total collagen synthesis and expression of collagen types I and III than the TDC group (*p* = 0.038, *p* = 0.048, *p* = 0.008) after PDGF-BB stimulation (Figure 2c,d). Collagen type II synthesis was not significantly affected in any group (Data not shown).

### 3.4. Osteogenic Differentiation

**Figure 3:**
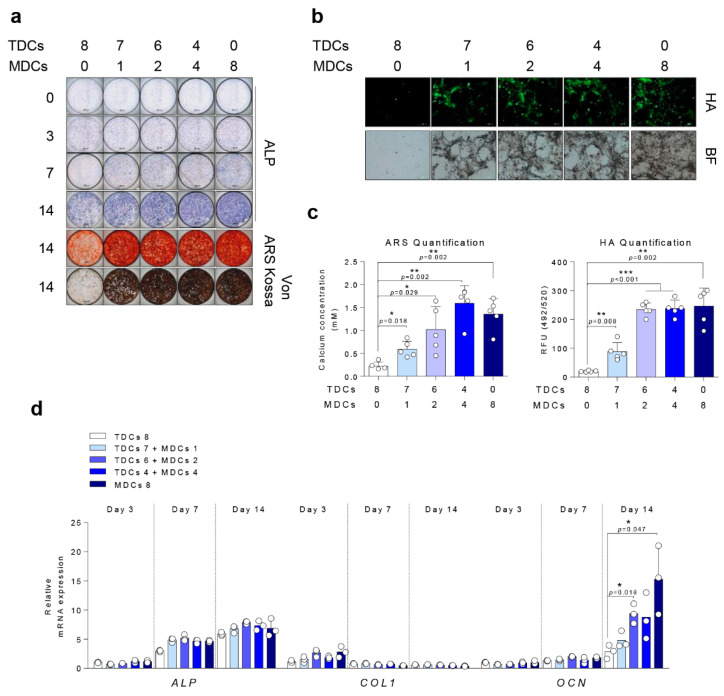
MDCs-enhanced bone mineralization of TDCs in response to osteogenic differentiation. A total of 8 × 10^3^ cells/well (tendon and/or muscle cells, as indicated) were cultured in osteogenic media for the indicated number of days and assessed by (**a**) ALP, ARS, and Von Kossa staining (scale bar = 200 µm) and (**b**) HA staining (scale bar = 500 µm). (**c**) Quantification of ARS and HA staining. (**d**) A total of 8 × 10^4^ cells (TDCs and/or MDCs, as indicated) were cultured in osteogenic media for the indicated number of days and analyzed by qPCR for differentiation markers. *, *p* < 0.05; **, *p* < 0.01; ***, *p* < 0.001. Data are presented as the mean ± SEM.

Osteogenic differentiation activities assessed by ALP, ARS, VON, and HA staining, and RT-qPCR were significantly higher in the MDC group than the TDC group and the proportionally increasing ratios of MDCs in the co-culture groups suggest that MDCs possess a stronger capacity for mineralization than TDCs (Figure 3).

### 3.5. Chondrogenic Differentiation

**Figure 4:**
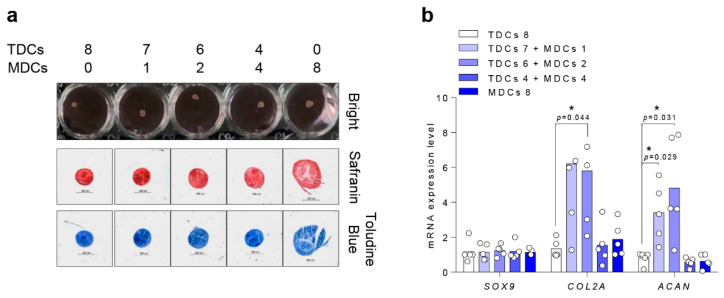
TDCs with a small population of MDCs contributes to induction of chondrocyte extracellular matrix genes under chondrogenic differentiation. A total of 2 × 10^5^ cells/well (TDCs and/or MDCs, as indicated) were cultured in chondrogenic medium for two weeks and analyzed by (**a**) safranin O and toluidine blue staining (scale bar = 200 µm) and (**b**) RT-qPCR for differentiation markers. *, *p* < 0.05. Data are presented as the mean ± SEM.

Chondrogenic pallet cultures showed successful ECM accumulation at the lining in all groups on day 14 of chondrogenesis. However, in the MDC group, the chondrogenic pallet was bigger and less stable in condensation compared with other groups (Figure 4a). Expression of cartilage-specific markers [36,37] including COL2 and ACAN was significantly increased in the 6:2 co-culture group compared with the TDC group (*p* = 0.044, *p* = 0.031) (Figure 4b). However, SOX9, which is another well-established chondrogenesis inducer [38,39], did not differ across any cell groups.

## 4. Discussion

This study demonstrated that muscle possesses a similarly strong capacity for cell proliferation and multilineage differentiation as tendon. At certain ratios of tendon and muscle co-cultures, cell viability and multilineage differentiation abilities were stronger than the single cell-type group of TDCs. Ligamentous and chondrogenic differentiation was most enhanced when a small population of MDCs was co-cultured with TDCs (6:2 co-culture group). Cell proliferation and osteogenic differentiation, on the other hand, were proportionally enhanced with increasing MDC population size.

Reconstruction with tendon graft (e.g., hamstring tendon) requires osteointegration of the tendon in the bone tunnel after primary fixation [10,11,12,13]. Many studies have reported changes in the healing of the graft-to-bone interface. Robert et al. [11] found that the fibrous interface was in contact with the bone, and Sharpey-like fibers started to be visible six months after ACL reconstruction with tendon graft. Another study by Eriksson et al. [12] found that osteointegration of the graft-to-bone interface occurred with continuity of collagen fibers of the tendon and bone one year after surgery. Furthermore, a study by Lazarides et al. [13] showed that collagen fibers were tightly interfaced into surrounding bone and there was abundant new bone formation. Studies commonly report an indirect insertion, defined as Sharpey-like fibers, that connect the tendon to the bone interface. However, normal ACL insertion occurs with fibrocartilaginous insertion, and no studies have successfully regenerated this fibrocartilaginous insertion at the tendon-bone interface [40,41].

Intra-articular graft remodeling has been extensively studied. The three different ligamentization stages are “early”, “remodeling”, and “maturation” [16]. Tendon grafts undergo histological rearrangement in response to biomechanical action and transform into a structure similar to that of a native ACL over time [14,15,16,17,18,19]. Numerous studies have attempted to evaluate graft ligamentization at six months post-ACL reconstruction, because this is a common timeframe for athletes to return to sports activities [18,42]. Although Howell et al. [17] showed that grafts can obtain sufficient stability as early as three months postoperatively, Hofbauer et al. [18] found that hamstring autografts did not show equal MRI signal intensity six months following ACL reconstruction compared with healthy ACLs, thus were concluded to still be immature. Similarly, Sanchez et al. [15] found that the graft undergoes ligamentization beyond one year, and Rougraff et al. [19] identified immature grafts, as defined by degeneration and hypercellularity on Magnetic resonance imaging (MRI) findings, up to three years following ACL reconstruction.

Sun et al. [43] studied the effect of muscle-preserved tendon graft on intra-articular healing after ACL reconstruction in a rabbit animal model and found that muscle left on tendon grafts accelerated alterations in the matrix structure, recellularization, and revascularization, thus promoting intra-articular healing and remodeling of the graft. Cuti et al. [22] examined the capacity of muscle-derived stem cells harvested from hamstring tendon, and found that muscle cells expanded faster, exhibited more ALP activity, and had higher expression of bone sialoprotein than tendon cells. Landry et al. [44] studied the periosteal response to skeletal trauma when the muscle was also injured and found that muscle injury increased proliferation in the periosteum and induction of osteoblasts during the early injury stages. Davis et al. [45] also studied how muscle contributes to fracture healing and found that muscle supplies osteoprogenitor cells and growth factors that play a role in muscle-bone interactions during inflammation. As tendon-to-bone interactions are often limited and intra-articular ligamentization occurs very slowly, additional strategies to enhance graft healing after ACL reconstruction are required. Our study findings—that MDCs co-cultured with TDCs at specific ratios enhance cell viability and are capable of multilineage differentiation—suggest that preservation of muscle on hamstring tendon grafts could offer a valuable source of stem cells that enhance biological healing following ACL reconstruction.

This study has several limitations. First, cells were cultured by multiple passages, and multiple passages may have caused different number of cells in different cell cycles [46,47]. Furthermore, dedifferentiation issue may be raised as tendon cells may become reversely differentiated during long culture periods [48]. Second, multilineage differentiation was induced by various growth factors, and this experimental environment does not fully reflect clinical reality. Third, co-culturing of TDCs and MDCs was performed to simulate the condition of preserved muscle remnants on hamstring tendon grafts, however, it is not clear whether such direct cell-to-cell contact would occur in reality. Fourth, the effect of remnant muscle tissue on the tendon graft’s biomechanical strength is unknown. However, because tendon graft thickness is uninfluenced by muscle, and a small volume of muscle preservation is recommended, a negative influence on biomechanical strength is unlikely. Finally, optimal ratios for co-culturing of TDCs and MDCs are unknown, and this issue should be addressed in the future study.

## 5. Conclusions

Ligamentous and chondrogenic differentiation was most enhanced when a small population of MDCs was co-cultured with TDCs (6:2 co-culture group). Cell viability and osteogenic differentiation were proportionally enhanced with increasing MDC population size. MDCs co-cultured with TDCs possess both the ability to enhance cell viability and differentiate into other cell lineages.

## Figures and Tables

**Table 1:** RT-qPCR primers used in the study.

Gene.	5′-Forward-3′	3′-Reverse-5′
Col1	ACATGCCGTGACTTGAGACTCA	GCCAGACGTGTTTCTTGTCCTT
Col2	GCCGGATCTGTGTCTGTGAC	TGTCCCTTTGGTCCTGGTTG
Col3	CTTCTCTCCAGCCGAGCTTC	CCAGTGTGTTTCGTGCAACC
Col6	CCTGGGCGTCAAAGTCTTCT	AGCACACTTGCTCCACGTTA
Col9	GCCCCAGATTCCCTGTCAAT	CCAACTTGTAAGCCACCTGC
Col10	CTGGACCGGCTGGAATTTCT	CGACCAGGAGCACCATATCC
Col11	ATCACAGGTGATCCCAAGGC	ACGATGTTTGCCTCATCTATCTG
ALP	CCAAGGACGCTGGGAAATCT	TATGCATGAGCTGGTAGGCG
SOX9	CTGAACGAGAGCGAGAAGCG	CCCGTTCTTCACCGACTTCC
ACAN	CTGGTCAGATGGACACCCCAT	CGTTTGTAGGTGGTGGCTGTG
TNC	GGTTGCTGGAGACTGTGGAA	AGGTTTTCCAGAAGGGGCAG
OCN	GATGGTCTCAAGCCCTGGTT	CCTCCCGTAGTGCTCCGATA
GAPDH	CAAGATCATCAGCAATGCC	CTGTGGTCATGAGTCCTTCC

## Data Availability

The data presented in this stud are available on request from the corresponding author.

## Supplementary Materials

The following are available online at https://www.mdpi.com/article/10.3390/cells10040740/s1. Figure S1. ALP staining, activity, and relative mRNA expression were relatively higher in MDC and TDC co-culture than in the TDC group.

## Abbreviations

ACLR: Anterior cruciate ligament reconstruction; TDCs, tendon-derived cells; MDCs, mus-cle-derived cells; PDGF-BB, Platelet-Derived Growth Factor-BB; ALP, Alkaline phosphatase; ARS, Alizarin Red S; HA, Hydroxyapatite; COL, Collagen; ACAN, aggrecan.

## References

[1] Duquin T.R., Wind W.M., Fineberg M.S., Smolinski R.J., Buyea C.M. (2009). Current trends in anterior cruciate ligament reconstruction. J. Knee Surg..

[2] Murray M.M., Martin S.D., Martin T.L., Spector M. (2000). Histological changes in the human anterior cruciate ligament after rupture. J. Bone Joint Surg. Am..

[3] Agung M., Ochi M., Yanada S., Adachi N., Izuta Y., Yamasaki T., Toda K. (2006). Mobilization of bone marrow-derived mesenchymal stem cells into the injured tissues after intraarticular injection and their contribution to tissue regeneration. Knee Surg. Sports Traumatol. Arthrosc..

[4] Menetrey J., Duthon V.B., Laumonier T., Fritschy D. (2008). “Biological failure” of the anterior cruciate ligament graft. Knee Surg. Sports Traumatol. Arthrosc..

[5] Tashiro Y., Gale T., Sundaram V., Nagai K., Irrgang J.J., Anderst W., Nakashima Y., Tashman S., Fu F.H. (2017). The Graft Bending Angle Can Affect Early Graft Healing After Anterior Cruciate Ligament Reconstruction: In Vivo Analysis With 2 Years’ Follow-up. Am. J. Sports Med..

[6] Jansson K.S., Costello K.E., O’Brien L., Wijdicks C.A., Laprade R.F. (2013). A historical perspective of PCL bracing. Knee Surg. Sports Traumatol. Arthrosc..

[7] Samuelsson K., Andersson D., Ahlden M., Fu F.H., Musahl V., Karlsson J. (2013). Trends in surgeon preferences on anterior cruciate ligament reconstructive techniques. Clin. Sports Med..

[8] Lee D.W., Shim J.C., Yang S.J., Cho S.I., Kim J.G. (2019). Functional Effects of Single Semitendinosus Tendon Harvesting in Anatomic Anterior Cruciate Ligament Reconstruction: Comparison of Single versus Dual Hamstring Harvesting. Clin. Orthop. Surg..

[9] Kyung H.S. (2019). Graft considerations for successful anterior cruciate ligament reconstruction. Knee Surg. Relat. Res..

[10] Lu H., Chen C., Xie S., Tang Y., Qu J. (2019). Tendon Healing in Bone Tunnel after Human Anterior Cruciate Ligament Reconstruction: A Systematic Review of Histological Results. J. Knee Surg..

[11] Robert H., Es-Sayeh J., Heymann D., Passuti N., Eloit S., Vaneenoge E. (2003). Hamstring insertion site healing after anterior cruciate ligament reconstruction in patients with symptomatic hardware or repeat rupture: A histologic study in 12 patients. Arthroscopy.

[12] Eriksson K., Kindblom L.G., Wredmark T. (2000). Semitendinosus tendon graft ingrowth in tibial tunnel following ACL reconstruction: A histological study of 2 patients with different types of early graft failure. Acta Orthop. Scand..

[13] Lazarides A.L., Eward W.C., Green K., Cardona D.M., Brigman B.E., Taylor D.C. (2015). Histological Evaluation of Tendon-Bone Healing of an Anterior Cruciate Ligament Hamstring Graft in a 14-Year-Old Boy. Am. J. Sports Med..

[14] Falconiero R.P., DiStefano V.J., Cook T.M. (1998). Revascularization and ligamentization of autogenous anterior cruciate ligament grafts in humans. Arthroscopy.

[15] Sanchez M., Anitua E., Azofra J., Prado R., Muruzabal F., Andia I. (2010). Ligamentization of tendon grafts treated with an endogenous preparation rich in growth factors: Gross morphology and histology. Arthroscopy.

[16] Claes S., Verdonk P., Forsyth R., Bellemans J. (2011). The “ligamentization” process in anterior cruciate ligament reconstruction: What happens to the human graft? A systematic review of the literature. Am. J. Sports Med..

[17] Howell S.M., Knox K.E., Farley T.E., Taylor M.A. (1995). Revascularization of a human anterior cruciate ligament graft during the first two years of implantation. Am. J. Sports Med..

[18] Hofbauer M., Soldati F., Szomolanyi P., Trattnig S., Bartolucci F., Fu F., Denti M. (2019). Hamstring tendon autografts do not show complete graft maturity 6 months postoperatively after anterior cruciate ligament reconstruction. Knee Surg. Sports Traumatol. Arthrosc..

[19] Rougraff B.T., Shelbourne K.D. (1999). Early histologic appearance of human patellar tendon autografts used for anterior cruciate ligament reconstruction. Knee Surg. Sports Traumatol. Arthrosc..

[20] Ahn G.Y., Nam I.H., Lee Y.H., Lee Y.S., Choi Y.D., Lee H.H., Hwang S.H. (2018). Factors Affecting the Extent of Graft Tendon Synovialization after Double-Bundle Anterior Cruciate Ligament Reconstruction: Based on Second-Look Arthroscopic Findings. Clin. Orthop. Surg..

[21] Kim S.G., Jung J.H., Song J.H., Bae J.H. (2019). Evaluation parameters of graft maturation on second-look arthroscopy following anterior cruciate ligament reconstruction: A systematic review. Knee Surg. Relat. Res..

[22] Cuti T., Antunovic M., Marijanovic I., Ivkovic A., Vukasovic A., Matic I., Pecina M., Hudetz D. (2017). Capacity of muscle derived stem cells and pericytes to promote tendon graft integration and ligamentization following anterior cruciate ligament reconstruction. Int. Orthop..

[23] Biz C., Crimi A., Fantoni I., Pozzuoli A., Ruggieri P. (2019). Muscle stem cells: What’s new in orthopedics?. Acta Biomed..

[24] Harrell C.R., Fellabaum C., Jovicic N., Djonov V., Arsenijevic N., Volarevic V. (2019). Molecular Mechanisms Responsible for Therapeutic Potential of Mesenchymal Stem Cell-Derived Secretome. Cells.

[25] Jo S., Wang S.E., Lee Y.L., Kang S., Lee B., Han J., Sung I.H., Park Y.S., Bae S.C., Kim T.H. (2018). IL-17A induces osteoblast differentiation by activating JAK2/STAT3 in ankylosing spondylitis. Arthritis Res. Ther..

[26] Park P.-R., Jo S., Jin S.-H., Kim T.-J. (2020). MicroRNA-10b Plays a Role in Bone Formation by Suppressing Interleukin-22 in Ankylosing Spondylitis. J. Rheum. Dis..

[27] Lee J.K., Jo S., Lee Y.L., Park H., Song J.S., Sung I.H., Kim T.H. (2020). Anterior cruciate ligament remnant cells have different potentials for cell differentiation based on their location. Sci. Rep..

[28] Molloy T., Wang Y., Murrell G. (2003). The roles of growth factors in tendon and ligament healing. Sports Med..

[29] Kraus A., Luetzenberg R., Abuagela N., Hollenberg S., Infanger M. (2017). Spheroid formation and modulation of tenocyte-specific gene expression under simulated microgravity. Muscles Ligaments Tendons J..

[30] Stoll C., John T., Endres M., Rosen C., Kaps C., Kohl B., Sittinger M., Ertel W., Schulze-Tanzil G. (2010). Extracellular matrix expression of human tenocytes in three-dimensional air-liquid and PLGA cultures compared with tendon tissue: Implications for tendon tissue engineering. J. Orthop. Res..

[31] Nam B., Park H., Lee Y.L., Oh Y., Park J., Kim S.Y., Weon S., Choi S.H., Yang J.H., Jo S. (2020). TGFbeta1 Suppressed Matrix Mineralization of Osteoblasts Differentiation by Regulating SMURF1-C/EBPbeta-DKK1 Axis. Int. J. Mol. Sci..

[32] Ehnert S., van Griensven M., Unger M., Scheffler H., Falldorf K., Fentz A.K., Seeliger C., Schroter S., Nussler A.K., Balmayor E.R. (2018). Co-Culture with Human Osteoblasts and Exposure to Extremely Low Frequency Pulsed Electromagnetic Fields Improve Osteogenic Differentiation of Human Adipose-Derived Mesenchymal Stem Cells. Int. J. Mol. Sci..

[33] Estes B.T., Diekman B.O., Gimble J.M., Guilak F. (2010). Isolation of adipose-derived stem cells and their induction to a chondrogenic phenotype. Nat. Protoc..

[34] Dominici M., Le Blanc K., Mueller I., Slaper-Cortenbach I., Marini F., Krause D., Deans R., Keating A., Prockop D., Horwitz E. (2006). Minimal criteria for defining multipotent mesenchymal stromal cells. The International Society for Cellular Therapy position statement. Cytotherapy.

[35] Mafi P., Hindocha S., Mafi R., Griffin M., Khan W.S. (2011). Adult mesenchymal stem cells and cell surface characterization—A systematic review of the literature. Open Orthop. J..

[36] De Mattei M., Fini M., Setti S., Ongaro A., Gemmati D., Stabellini G., Pellati A., Caruso A. (2007). Proteoglycan synthesis in bovine articular cartilage explants exposed to different low-frequency low-energy pulsed electromagnetic fields. Osteoarthr. Cartil..

[37] Ciombor D.M., Lester G., Aaron R.K., Neame P., Caterson B. (2002). Low frequency EMF regulates chondrocyte differentiation and expression of matrix proteins. J. Orthop. Res..

[38] Diederichs S., Gabler J., Autenrieth J., Kynast K.L., Merle C., Walles H., Utikal J., Richter W. (2016). Differential Regulation of SOX9 Protein During Chondrogenesis of Induced Pluripotent Stem Cells Versus Mesenchymal Stromal Cells: A Shortcoming for Cartilage Formation. Stem. Cells Dev..

[39] Jiang X., Huang X., Jiang T., Zheng L., Zhao J., Zhang X. (2018). The role of Sox9 in collagen hydrogel-mediated chondrogenic differentiation of adult mesenchymal stem cells (MSCs). Biomater. Sci..

[40] Genin G.M., Thomopoulos S. (2017). The tendon-to-bone attachment: Unification through disarray. Nat. Mater..

[41] Rossetti L., Kuntz L.A., Kunold E., Schock J., Muller K.W., Grabmayr H., Stolberg-Stolberg J., Pfeiffer F., Sieber S.A., Burgkart R. (2017). The microstructure and micromechanics of the tendon-bone insertion. Nat. Mater..

[42] Ma Y., Murawski C.D., Rahnemai-Azar A.A., Maldjian C., Lynch A.D., Fu F.H. (2015). Graft maturity of the reconstructed anterior cruciate ligament 6 months postoperatively: A magnetic resonance imaging evaluation of quadriceps tendon with bone block and hamstring tendon autografts. Knee Surg. Sports Traumatol. Arthrosc..

[43] Sun L., Hou C., Wu B., Tian M., Zhou X. (2013). Effect of muscle preserved on tendon graft on intra-articular healing in anterior cruciate ligament reconstruction. Knee Surg. Sports Traumatol. Arthrosc..

[44] Landry P.S., Marino A.A., Sadasivan K.K., Albright J.A. (2000). Effect of soft-tissue trauma on the early periosteal response of bone to injury. J. Trauma.

[45] Davies O.G., Grover L.M., Eisenstein N., Lewis M.P., Liu Y. (2015). Identifying the Cellular Mechanisms Leading to Heterotopic Ossification. Calcif. Tissue Int..

[46] Mazzocca A.D., Chowaniec D., McCarthy M.B., Beitzel K., Cote M.P., McKinnon W., Arciero R. (2012). In vitro changes in human tenocyte cultures obtained from proximal biceps tendon: Multiple passages result in changes in routine cell markers. Knee Surg. Sports Traumatol. Arthrosc..

[47] Yao L., Bestwick C.S., Bestwick L.A., Maffulli N., Aspden R.M. (2006). Phenotypic drift in human tenocyte culture. Tissue Eng..

[48] Dede Eren A., Vasilevich A., Eren E.D., Sudarsanam P., Tuvshindorj U., de Boer J., Foolen J. (2020). Tendon-Derived Biomimetic Surface Topographies Induce Phenotypic Maintenance of Tenocytes In Vitro. Tissue Eng. Part A.

