# Supplementary material for: Effect of Muscle Cell Preservation on Viability and Differentiation of Hamstring Tendon Graft In Vitro
Source: Cells. 2021 Mar 27;10(4):740. doi: 10.3390/cells10040740 (PMC8065441; doi:10.3390/cells10040740)
Supplement: Supplementary file 1 [file cells-10-00740-s001.pdf]

Supplemental figure

Effect of Muscle Cell Preservation on Viability and Differentiation of Hamstring Tendon Graft In Vitro

Jin Kyu Lee, Sungsin Jo, Young Lim Lee, Subin Weon, Jun-Seob Song, Il-Hoon Sung, Tae-Hwan Kim

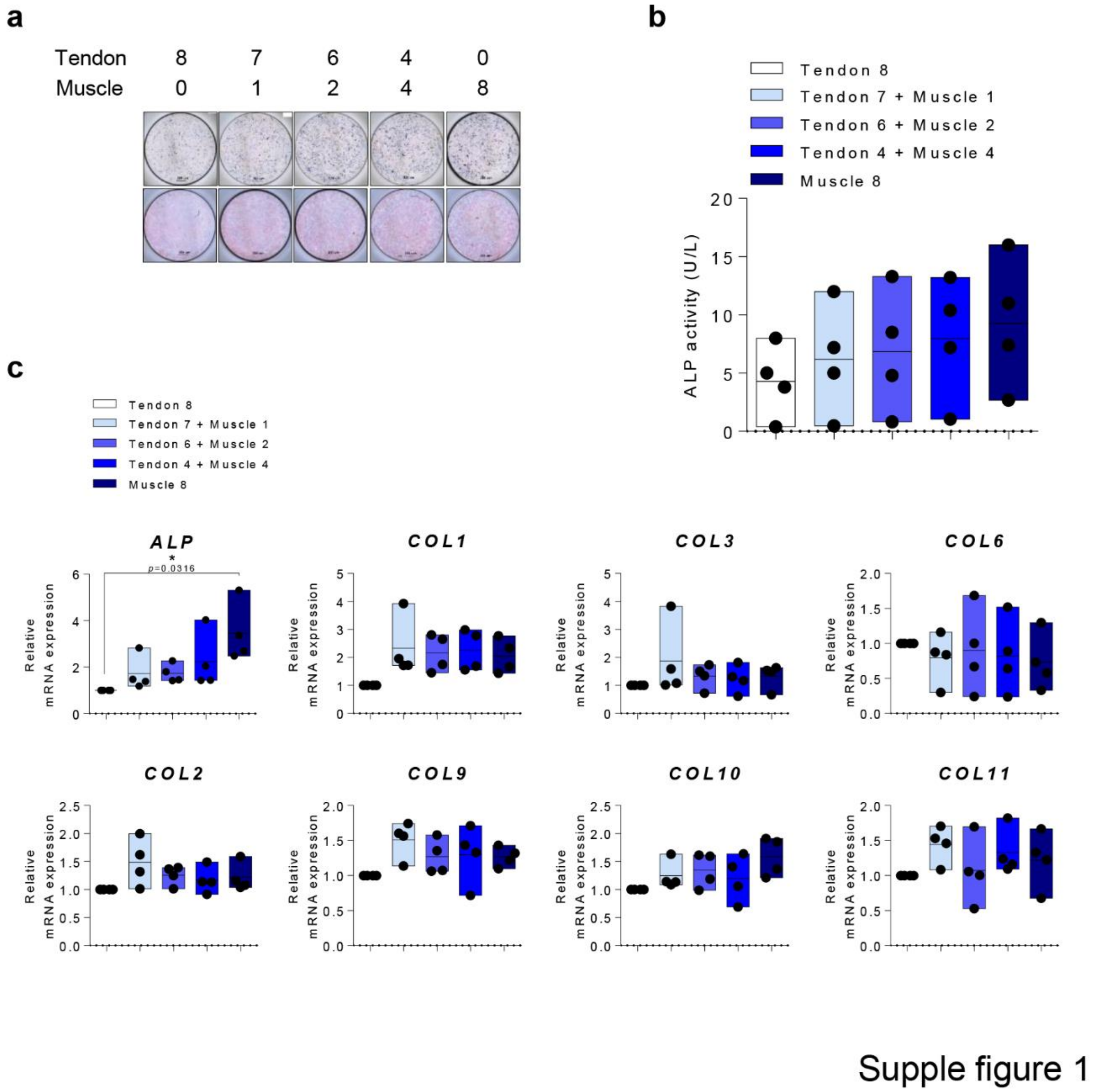

Figure 1. ALP staining, activity, and relative mRNA expression were relatively higher in MDC and TDC co-culture than in the TDC group. A total of 8e10³ cells/well (tendon and/or muscle cells, as indicated) were cultured AND assessed by (A) ALP staining (scale bar=200μm), (B) ALP activity, and (C) qPCR for collagen gene expressions.
